# Nano-Enabled Insecticides for Efficient Pest Management: Definition, Classification, Synergistic Mechanism, and Safety Assessment

**DOI:** 10.3390/nano15131050

**Published:** 2025-07-06

**Authors:** Ying Wei, Jingyi Chen, Min Dong, Meizhen Yin, Jie Shen, Le Gao, Shuo Yan

**Affiliations:** 1 State Key Laboratory of Agricultural and Forestry Biosecurity, MARA Key Laboratory of Surveillance and Management for Plant Quarantine Pests, College of Plant Protection, China Agricultural University, Beijing 100193, China; weiyingdawn@126.com (Y.W.); chenjingyi@cau.edu.cn (J.C.); dongmin@cau.edu.cn (M.D.); shenjie@cau.edu.cn (J.S.); 2 State Key Laboratory of Chemical Resource Engineering, Beijing Laboratory of Biomedical Materials, Beijing University of Chemical Technology, Beijing 100029, China; yinmz@mail.buct.edu.cn; 3 Department of Horticulture, Beijing Vocational College of Agriculture, Beijing 102442, China

**Keywords:** green pest management, nano-insecticide, nano-delivery system, synergistic effect, safety assessment

## Abstract

The widespread use of pesticides plays a vital role in safeguarding crop yields and ensuring global food security. However, their improper application has led to serious challenges, including environmental pollution, pesticide residues, and increasing insect resistance. Traditional chemical pesticides are no longer sufficient to meet the demands for sustainable modern agriculture. Recent advances in nanotechnology offer innovative strategies for improving pesticide delivery, bioavailability, and selectivity. This review systematically summarizes the current progress in nano-insecticides, including their definitions, classification, preparation techniques, synergistic mechanisms, insecticidal performance, and safety evaluation. In addition, emerging strategies, such as multi-stimuli responsive systems, co-delivery with multiple agents or genetic materials, and integration with biological control, are discussed. Finally, future perspectives are proposed to guide the design/development of intelligent, efficient, and eco-friendly nano-insecticides for sustainable pest management in modern agriculture.

## 1. Introduction

Agricultural production serves as a critical foundation for human survival and societal development by providing essential food resources [1,2]. Nevertheless, agricultural practices have continuously faced significant threats from various plant diseases and insect infestations, which severely compromise crop health and productivity [3]. Globally, insect pests alone account for up to 40% of annual crop losses, resulting in substantial economic damages estimated at approximately USD 220 billion each year [4,5]. The development and widespread application of pesticides have markedly improved both crop quality and yield [6,7]. It is estimated that approximately one-third of global agricultural output is safeguarded by pesticides, highlighting their indispensable role in ensuring food security [3]. The indiscriminate and unscientific application of these chemicals has led to persistent pesticide residues in agricultural products, soil, and groundwater, consequently raising significant public concerns regarding environmental pollution and food safety [8,9,10]. Additionally, excessive pesticide application accelerates the evolution and selection of resistant pest populations and leads to notably low utilization efficiency, presenting a major and persistent challenge for effective plant protection [11,12]. Publications have indicated that more than 90% of applied pesticides do not reach the target organisms, with only about 5% effectively contacting the pests and less than 0.1% ultimately being absorbed and exerting the intended biological effects [13,14,15]. Consequently, traditional pesticides are increasingly inadequate for meeting contemporary sustainable agricultural needs, particularly under the pressures of global population growth and climatic deterioration [16,17,18]. Thus, it is imperative to develop more efficient and eco-friendly pest control strategies.

In this context, the incorporation of nanotechnology into agriculture offers promising avenues for improving pest management, giving rise to the innovative concept of “nano-pesticides” [19,20]. Recognized by the International Union of Pure and Applied Chemistry (IUPAC) in 2019 as one of the top ten emerging chemical technologies [21], nano-pesticides represent a transformative advancement over traditional agrochemicals. Nano-pesticides are broadly defined as pesticide formulations engineered with nanoscale materials or nanotechnology principles. Compared to conventional pesticides, nano-pesticides offer several advantages: (i) responsiveness to environmental stimuli; (ii) efficient target-specific delivery; (iii) greater efficacy against pests, diseases, and weeds; and (iv) alleviation of environmental pollution and non-target toxicity [22,23]. This review systematically examines nano-insecticides by discussing their definition, classification, preparation methods, synergistic mechanisms, pest control efficacy, and environmental safety considerations. Furthermore, we explore future trends in nano-insecticide development, with the overarching aim of providing comprehensive theoretical guidance for in-depth research and diversified applications in the agricultural field.

## 2. Definition and Connotation of Nano-Pesticides

Currently, there is no internationally unified definition of nano-pesticides. Over the past decade, debates have persisted regarding whether nano-pesticides should be defined strictly based on particle size criteria. Kah and Hofmann [22] argued that setting a rigid threshold of less than 100 nm as the evaluation criterion is overly simplistic and cannot comprehensively encompass the diversity of nano-formulations. They proposed that pesticides operating at the nanoscale (below 1000 nm) or exhibiting novel properties attributed to nanoscale dimensions should be categorized as nano-pesticides. Subsequently, Wang et al. [4] further refined this perspective, suggesting that pesticides with particle sizes below 500 nm are more representative of typical nanoscale characteristics, thereby narrowing the size range for defining nano-pesticides. More recently, the agricultural industry standard “Rules for Drafting Specifications for Nano-Pesticide Products,” led by Professor Lidong Cao from the Institute of Plant Protection, Chinese Academy of Agricultural Sciences, was officially issued by the Ministry of Agriculture and Rural Affairs of the People’s Republic of China [24]. This standard defines nano-pesticides as formulations in which the active ingredient (AI) is stabilized in a nanoscale dispersed state within the formulation and/or the dispersion system during application through the use of nanoscale preparation technologies. Notably, the standard refrains from imposing a universal fixed size limit but specifies size restrictions tailored to three distinct categories of nano-pesticides [25]:

(i) Nano-emulsion, referring to oil-in-water (O/W) emulsions in which water-insoluble AIs are finely dispersed into nanoscale particles (1–100 nm) by surfactants and other functional agents, thereby forming kinetically stable nanoparticle-based emulsions. For example, Abd-Elnabi et al. [26] formulated nano-emulsions using essential oils from *Portulaca oleracea* and *Rosmarinus officinalis*, achieving mean particle sizes of 26.67 nm and 97.36 nm, respectively. These nanoscale emulsions exhibit substantially greater insecticidal activity against *Aphis gossypii*, *Spodoptera littoralis*, and *Tetranychus urticae*, with LC_50_ values significantly lower than those of their bulk oil counterparts. The enhanced efficacy is attributed to the smaller particle size, which can increase foliar coverage, improve wettability, and enhance cuticular penetration.
(ii) Nano-suspension concentrate: A suspension formulation where the AI and solid excipients are present as nanosized particles (1–300 nm), stabilized in water via nano-techniques. For instance, Ding et al. [27] formulated a chlorantraniliprole nanosuspension with a mean particle size of 56 nm for controlling *Cnaphalocrocis medinalis*. Compared to a commercial suspension concentrate, the nano-suspension exhibits superior dispersibility, foliar wettability, and leaf retention, leading to improved bioavailability and efficacy. The formulation achieves equivalent control efficacy, even at a 40% reduced dose, and remains physically stable during storage at 25 °C.
(iii) Nano-water dispersible granule (nano-WDG): A granule formulation has been prepared via nanotechnological processes that disintegrates in water to release nanoscale solid particles (1–300 nm). Li et al. [28] reported a solid nano-dispersion of emamectin benzoate with an average particle size of 17 nm and 50% loading content using PEG 4000 and surfactants through a melt-fusion method. This nano-WDG exhibits 1.8-fold higher insecticidal activity against *Spodoptera exigua* compared to commercial WDG, highlighting its improved dispersibility and bio-efficacy.

This evolving standard represents a significant milestone in establishing a scientifically grounded and practically applicable classification framework for nano-pesticides, providing clearer guidance for both regulatory oversight and technological development.

## 3. Classification and Preparation Method of Nano-Insecticides

Nano-insecticides can be mainly classified into two types. The first type (Type I) comprises nano-insecticides in which the nanomaterials themselves serve as the AIs, possessing inherent pesticidal activity due to their physicochemical properties at the nanoscale. The second type (Type II) refers to nano-delivery systems in which nanomaterials are employed as carriers to encapsulate, load, or stabilize conventional AIs. More specifically, Type I nano-insecticides can be further divided according to the physicochemical nature of the nanoscale AIs. These include metal-based nano-insecticides, such as nano-copper or nano-silver, and non-metallic nano-insecticides, such as nanoscale carbon materials or silicon-based particles. In contrast, Type II nano-insecticides are classified based on the nature of the nanocarriers used. They generally fall into four categories: (i) inorganic carriers, (ii) organic carriers, (iii) inorganic–organic hybrid carriers, and (iv) small-molecule-based carriers [25]. These classifications provide a comprehensive framework for understanding the diversity and formulation logic of nano-insecticides, which is critical for evaluating their behavior, functionality, and potential environmental implications.

### 3.1. Type I Nano-Insecticides

#### 3.1.1. Metal-Based Nano-Insecticides

Metal-based nano-insecticides are composed of various metallic elements, including gold, silver, copper, iron, palladium, and nickel, as well as metal oxides, such as zinc oxide, titanium dioxide, aluminum oxide, iron oxide, copper oxide, magnesium oxide, and cadmium oxide. These nanomaterials exhibit intrinsic pesticidal activity and have been employed against a wide range of agricultural, veterinary, and public health pests, including *Spodoptera litura*, *Hyalomma anatolicum*, *Aedes aegypti*, etc. [29,30,31].

The preparation methods for metal-based nano-insecticides can be broadly categorized into three main approaches: physical methods, chemical synthesis, and green-chemistry synthesis. Physical methods typically involve top-down techniques such as laser ablation, high-energy ball milling, and physical vapor deposition to reduce bulk materials to nanoscale dimensions. In chemical synthesis, metal ions such as Ag^+^ can be reduced to metallic nanoparticles using various organic or inorganic agents based on redox reactions [32]. For example, copper oxide nanoparticles (CuO NPs) can be synthesized via chemical precipitation and subsequently incorporated into reduced graphene oxide (rGO) composites to form rGO-CuO NPs [33]. Similarly, Silva et al. [34] successfully synthesized zinc oxide quantum dots through a sol-gel method for effective control of *A. aegypti*. Green-chemistry synthesis aligns with the principles of chemical synthesis but utilizes biological entities, such as plant extracts, bacteria, or fungi, as environmentally benign reducing and stabilizing agents [35]. For instance, Bharani and Namasivayam [36] synthesized stable and uniform silver nanoparticles using pomegranate peel extract, which demonstrated significant larvicidal activity against *S. litura.* Likewise, *Beauveria bassiana*, an entomopathogenic fungus, was employed to biosynthesize silver nanoparticles, offering a green and effective method for mosquito control [37].

In addition to their synthesis advantages, metal-based nanoparticles exert pesticidal effects through multiple mechanisms. Silver nanoparticles (AgNPs) can adhere to and penetrate insect cuticles, physically damaging tissues and enhancing desiccation [38]. They also release Ag^+^ ions that interfere with respiratory enzymes and induce excessive reactive oxygen species (ROS), leading to oxidative stress and apoptosis [39]. Furthermore, AgNPs disrupt antioxidant enzyme activity and induce genotoxic effects that impair insect metabolism. CuO NPs similarly act by triggering ROS generation, causing lipid peroxidation, and increasing membrane permeability through protein–lipid interactions [40,41]. The third instar larvae of *S. litura* show 100% mortality at 144 h after exposure to 500 ppm ZnO NPs [42]. Collectively, these multi-targeted biochemical and physiological disruptions enable metal-based nano-insecticides to exert broad-spectrum and highly effective insecticidal activity.

#### 3.1.2. Non-Metallic Nano-Insecticides

Non-metallic nano-insecticides primarily consist of active nano-inorganic materials, such as nano-silica, as well as individual nanoscale AIs without carrier systems [25]. Shoaib et al. [43] synthesized nano-silica with an average particle size of 25 nm using a sol-gel method, and powder application at 1 mg/cm^2^ resulted in 85% mortality of *Plutella xylostella*. Green synthesis methods utilizing plant-derived materials were also employed to prepare silica nanoparticles with diameters below 100 nm [44,45]. Gui et al. [46] adopted a template-assisted synthesis approach using polystyrene as a template and reacting hexadecyltrimethylammonium bromide with tetraethyl orthosilicate, followed by calcination, to produce hollow mesoporous silica nanoparticles (HMS) with an approximate size of 200 nm. Nano-silica adhere to the insect cuticle, absorb protective lipids, disrupt the hydrophobic barrier, and cause desiccation and death, effectively “sanding down” the exoskeleton [47,48]. In addition, individual nanoscale AIs represent a category of carrier-free nano-pesticides designed through prodrug concepts and molecular self-assembly for plant protection [49,50,51]. The carrier-minimized systems like Asp–SSD–APG (aspartic acid–spinosad) have demonstrated enhanced immediate toxicity against *Musca domestica*, with reduced non-target toxicity via inhibition of the detoxifying enzymes SOD, CAT, and GST [52].

### 3.2. Type II Nano-Insecticides

#### 3.2.1. Nano-Insecticides Based on Inorganic Carriers

Encapsulation or loading of pesticides into nanocarriers is currently the most widely reported and applied strategy in nano-pesticide formulation research [53,54,55]. Common inorganic carriers, including mesoporous silica, nano-clay, nano-calcium carbonate, graphene oxide, biochar, and boron nitride, have been extensively utilized due to their high surface area, tunable pore structures, and excellent physicochemical stability [56,57]. Recently, MSNs mitigate the rapid degradation and volatilization of encapsulated agents, maintaining effective concentrations over extended periods. For example, Li et al. [58] demonstrated that β-cyhalothrin-loaded MSNs exhibited sustained release in response to foliar pH changes, extending the larvicidal activity against *Ostrinia furnacalis* for over 7 days.

Nanoscale calcium carbonate (CaCO_3_ NPs) is particularly suitable for acidic-triggered release systems. It is typically synthesized via controlled precipitation—mixing CaCl_2_ and Na_2_CO_3_ under specific surfactant ratios in reverse-phase microemulsions—yielding nanocrystals (50–200 nm) with high loading efficiency (≈20%) [59]. Notably, as dry particle films, CaCO_3_ NPs coat insect attachment organs (tarsi, pulvilli), disrupting adhesion and increasing detachment [60]. Furthermore, graphene oxide (GO) has been explored as a multifunctional carrier for loading beta-cyfluthrin, monosultap, and imidacloprid, significantly enhancing their insecticidal activity against lepidopteran pests [61]. GO’s layered structure enhances insecticide uptake by providing greater adhesion to insect cuticles and foliage. Laboratory trials demonstrated a 1.5–1.8-fold increase in acaricidal activity against *T. urticae* using GO-loaded insecticides due to better coating and slow environmental release [62].

#### 3.2.2. Nano-Insecticides Based on Organic Carriers

The types of organic polymers for preparing nano-pesticides are numerous, including natural polymers (chitosan, sodium alginate, cyclodextrin, etc.) and synthetic polymers (polyesters, polyureas, polyurethanes, block copolymers, etc.) [25,63]. These materials serve as carriers to encapsulate AIs within nanoscale delivery systems, such as nanocapsules, nanomicelles, and nanogels [63]. Organic carrier systems offer distinct advantages by enabling the controlled release of insecticides in response to specific environmental triggers, such as pH, temperature, or light exposure [23,64]. Additionally, they help minimize pesticide loss through leaching and volatilization, thereby reducing environmental contamination and improving application efficiency [65,66].

Common preparation techniques include interfacial polymerization, emulsion polymerization, nanoprecipitation, and molecular self-assembly [67,68]. For natural polymers, bioadhesive polymers like chitosan can penetrate insect midgut and respiratory membranes, increasing internal AI concentrations. For instance, β-myrcene-loaded chitosan nanoparticles were found to accumulate in the larval gut of *Aedes aegypti* and achieve 100% mortality at 238 mg/L [69]. For synthetic polymers, a series of LC-loaded poly (octyl acrylate) nanogel formulations (LONFs) (particle size ~150 nm) were prepared using microemulsion polymerization, achieving excellent physical stability and bio-efficacy [70]. Zhang et al. [71] synthesized camptothecin (CPT)-based micelles via self-assembly with polyethylene glycol (PEG), demonstrating potent acaricidal activity against *Tetranychus cinnabarinus*. In another example, a star polymer (SPc) was employed to spontaneously encapsulate dinotefuran through hydrogen bonding and Van der Waals interactions, dramatically increasing its insecticidal toxicity against *Myzus persicae* [72].

#### 3.2.3. Nano-Insecticides Based on Inorganic–Organic Hybrid Carriers

Inorganic–organic hybrid carriers are constructed by integrating inorganic frameworks with organic components, either through physical modification or covalent/coordination interactions. A common approach involves modifying inorganic materials, such as mesoporous silica nanoparticles (MSNs), with biocompatible polymers like chitosan or dopamine to enhance functionality and responsiveness. Alternatively, hybrid systems can also be constructed via the self-assembly of inorganic compounds and organic ligands through coordination bonding, with metal–organic frameworks (MOFs) serving as a representative class [25]. For instance, Xu et al. [73] developed dopamine-functionalized MSNs and subsequently grafted a stimuli-responsive copolymer (PVNM) onto their surfaces via conventional free radical polymerization. The polymerization utilized azodiisobutyronitrile (AIBN) as an initiator and included monomers such as tri (ethylene glycol) methyl ether methacrylate (MEO3MA), UV-crosslinkable monomer (VM), and N-vinyl caprolactam (NVCL). The resulting MSNs-g-PVNM nanocarriers, with an average particle size of approximately 150 nm, achieved a high loading efficiency (40.6%) for the model insecticide imidacloprid. Under ambient light/temperature, the PVNM cap remains intact to minimize premature insecticide leaching, and only target-specific environmental conditions trigger insecticide delivery to improve foliar retention. In another example, Zhang et al. [74] synthesized a dual-responsive MOF, zeolitic imidazolate framework-8 (ZIF-8), capable of responding to both pH changes and enzymatic activity (amylase) via an in situ crystal growth method. Thiacloprid was efficiently encapsulated into the ZIF-8 matrix through a one-pot self-assembly strategy, enabling smart and stimuli-responsive release for the targeted control of aphids. These hybrid systems offer structural tunability, high loading capacity, and stimulus responsiveness, positioning them as promising platforms for next-generation precision insecticide delivery.

#### 3.2.4. Nano-Insecticides Based on Small Molecules

This category of nano-insecticides involves the spontaneous self-assembly of AIs with small molecules via noncovalent interactions, leading to the formation of nanoscale delivery systems without the need for traditional nanocarriers [25,50]. These interactions may include electrostatic attraction, hydrogen bonding, or other noncovalent forces, depending on the chemical structures of the AIs and small molecules. For instance, Tian et al. [75] utilized electrostatic interactions between spinosad and sulfanic acid (SSD-SA) to fabricate spinosad nanoparticles with an average diameter of approximately 7 nm, and this nanoscale formulation displays the markedly stronger insecticidal activity. The SSD–SA NPs exhibit strong positive zeta potential (+47.8 mV), which improves their affinity to negatively-charged insect cuticle and gut surfaces, thereby enhancing insecticide uptake to increase insecticidal activity.

A recent and representative example is provided by Qi et al. [76] for nano-fungicides, and two fungicidal molecules—fenhexamid (FHA) and prochloraz (PRO)—were co-assembled into FHA–PRO nanoparticles (~80–120 nm) via electrostatic and hydrophobic interactions in aqueous conditions. This carrier-free design effectively converts the AIs into self-assembled nanodispersions. Similarly, the co-delivery insecticidal nanoparticles based on thiamethoxam and lambda-cyhalothrin have been fabricated utilizing a co-assembly strategy. The resulting co-assembled nanoparticles have pH-responsive release properties, which have excellent synergistic biological activity against *Aphis gossypii* [77]. Most notably, Shangguan et al. [50] recently introduced the innovative concept of carrier-minimized nano-pesticides. This strategy employs prodrug design and molecular self-assembly to produce nanoscale formulations with minimal or no reliance on conventional carriers. The approach offers multiple advantages, including energy-efficient synthesis, high AI loading, and broad potential for structural functionalization. As such, it represents a highly promising direction for the development of green and sustainable nano-insecticide technologies.

## 4. Advantages of Nano-Insecticides

**Figure 1:**
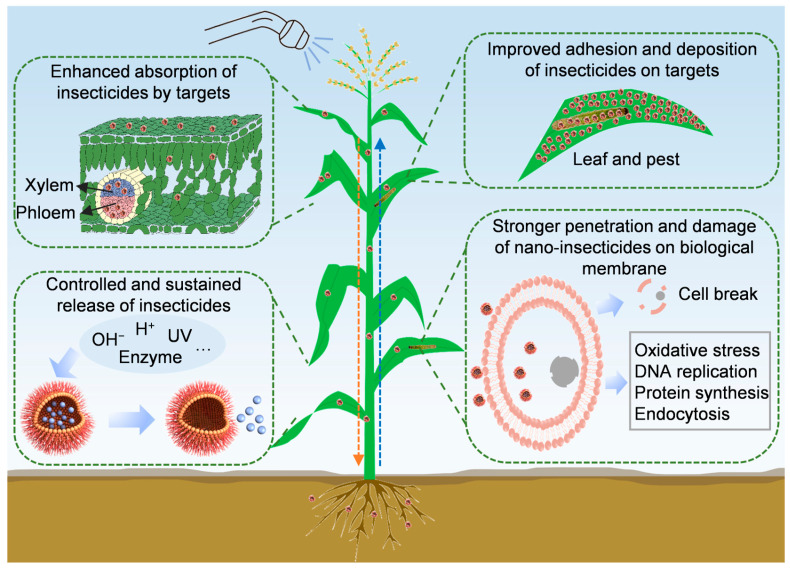
Synergistic mechanisms of nano-insecticides for controlling insect pests.

Nano-insecticides exhibit a range of advantageous physicochemical properties, including nanoscale size effects, stimuli responsiveness, controlled release behavior, and enhanced interfacial activity [19,78]. These characteristics collectively contribute to their superior efficacy in controlling insect pests (Figure 1).

### 4.1. Improved Adhesion and Deposition of Insecticides on Targets

Efficient adhesion and deposition of insecticides on target surfaces are fundamental to effective dose delivery and pest control outcomes. In this context, targets are typically classified into two categories: “large targets,” referring to the crops intended for protection, and “small targets,” denoting the pests or pathogens targeted for suppression [79,80]. However, pesticide retention on plant foliage is often limited due to the hydrophobic nature and micro/nano-structural complexity of plant surfaces (primarily driven by wax layers), which result in droplet bounce, fragmentation, and surface runoff [81,82,83]. Nano-pesticides, by virtue of their small particle size, superior interfacial activity, and tunable physicochemical properties, can significantly enhance droplet adhesion, wetting, and deposition on leaf surfaces, thereby improving pesticide utilization efficiency [84,85]. For instance, Yu et al. [86] designed nano-formulations of abamectin and azoxystrobin modified with tannic acid, which established hydrogen bonding with leaf surface microstructures, thereby increasing insecticide retention. Similarly, nanoscale formulations exhibited enhanced foliar stability, wettability, and deposition capacity, leading to improved insecticidal activity against aphids [85]. For instance, emamectin benzoate was formulated with nanoparticles composed of DSPE-PEG2000-NH2 by the co-solvent method, and the resulting nanoparticles had a regular distribution, spherical shape, good leaf wettability, and strong adhesion on maize foliage [87]. In another study, binary nanocarrier systems were constructed to load acetamiprid into nanomicelles, achieving over 70% retention on leaf surfaces [88]. The SPc-loaded avermectin nanoparticles (~100 nm) were shown to reduce contact angle, enlarge contact area, and significantly enhance contact toxicity against *M. persicae* [89]. In addition, nanoscale silicon dioxide was reported to effectively deposit on the cuticle of *P. xylostella*, physically damaging the exoskeleton and obstructing spiracles to ultimately cause insect death [43].

### 4.2. Enhanced Absorption of Insecticides by Targets

Nano-formulated insecticides can significantly enhance the absorption and translocation of AIs within plant tissues, thereby improving control efficacy against piercing–sucking pests, subterranean insects, and stem-boring pests [90,91,92]. This enhancement is primarily attributed to the nanoscale dimensions and surface modification of nanocarriers, which facilitate the penetration through the plant epidermis, vascular tissues, and insect integuments. For instance, it was reported that the incorporation of SPc into imidaclothiz formulations markedly improves its plant uptake, resulting in elevated aphid mortality [93]. Similarly, hollow mesoporous silica nanoparticles (HMSNs) were shown to promote the uptake and systemic movement of rotenone in cucumber plants [94]. Wang et al. [95] developed nano-delivery systems at 50 nm, which can reach vascular tissues through the symplastic pathway to allow for the long-distance transport of acetamiprid. Su et al. [96] developed a bovine serum albumin-based nanocarrier to encapsulate thiacloprid, which enabled efficient xylem transport within tree trunks and enhanced insecticide penetration across the insect cuticle, thereby increasing the toxicity by 25.9%. Moreover, nanoscale metallic particles (e.g., Au, CuO, ZnO, MgO, etc.) can be readily absorbed by insect tissues, either through cuticular penetration or disruption of respiratory structures such as siphons, ultimately resulting in insect death [30].

### 4.3. Controlled and Sustained Release of Insecticides

In field environments, insecticide efficacy is often compromised due to a range of abiotic and biotic factors, including sunlight, ultraviolet (UV) radiation, pH variation, and enzymatic degradation, which collectively accelerate the decomposition of AIs and reduce their effective duration [97]. Nano-formulated insecticide delivery systems can mitigate these limitations by enabling controlled, sustained, and stimulus-responsive release of AIs, thereby improving their stability, bioavailability, and long-term field performance. For instance, CPT is a potent botanical insecticide, but its insecticidal activity is severely limited in the alkaline midgut of lepidopteran pests [98]. To overcome this limitation, a pH- and redox-responsive nano-insecticide (CPT@Zein-FA) was designed using plant-derived zein protein and ferulic acid as carriers, allowing for the targeted release of CPT under physiological conditions within insect bodies. In another study, a temperature-responsive mixed micelle (MMs–Pys–7) was developed for the controlled release of pyrethrins, and a release mode consistent with larval population variations with temperature was successfully achieved [99]. Similarly, a dinotefuran supramolecularly bonded layered double hydroxide (D-LDH) nano-insecticide was designed to improve the controlled release properties and insecticidal activity against *A. gossypii* in cucumber and cotton plants [100]. These examples demonstrate how nano-enabled sustained-release strategies can effectively extend the bioactivity window of insecticides while reducing environmental losses and application frequency.

### 4.4. Stronger Penetration and Damage of Nano-Insecticides on Biological Membrane

To exert their bioactivity, insecticides must overcome a series of biological barriers in pests, including the exoskeleton, cellular membranes, and organelle membranes, in order to reach their intracellular molecular targets [15,101]. Owing to their nanoscale size and enhanced surface properties, nano-insecticides exhibit superior penetration capabilities through these barriers, allowing for them to more effectively interfere with key physiological and biochemical processes in insect pests [29]. One common mechanism involves the induction of oxidative stress. Nano-pesticides can elevate reactive oxygen species (ROS), particularly superoxide anions, thereby disrupting redox homeostasis and leading to insect death and fungal inhibition [102,103]. Jiang et al. [104] formulated a nanoscale thiamethoxam delivery system using SPc, which significantly upregulated genes associated with transmembrane transport in *Spodoptera frugiperda*, resulting in a 27.5% increase in mortality. Other studies demonstrated that SPc can stimulate clathrin-mediated endocytosis in insects by activating clathrin-related genes, thereby enhancing the intracellular delivery and cytotoxicity of AIs [105,106]. Beyond intracellular delivery, some nano-insecticides act by physically disrupting insect biomembranes. For example, chlorfenapyr-loaded MOF nanoparticles were reported to damage the peritrophic membrane in *S. frugiperda*, substantially enhancing insecticidal efficacy [107]. Similarly, oral administration of certain nanocarriers resulted in membrane disruption and lysosomal dysfunction in gut tissues of *Harmonia axyridis*, as evidenced by the downregulation of genes related to membrane integrity and lysosome function [108]. In addition to improved membrane permeability, nano-insecticide formulations may also modulate the mode of action of conventional insecticides, thereby expanding their insecticidal spectrum and enhancing their versatility in integrated pest management strategies [101].

## 5. Insecticidal Performance of Nano-Insecticides

Despite the diverse acting modes provided by nanocarriers, the ultimate goal of nano-insecticide development lies in achieving enhanced field efficacy and reduced insecticide application rates. Nanocarrier systems have demonstrated significant potential to enhance the insecticidal performance of AIs, often enabling low application dosage while maintaining or improving control efficacy [109]. Numerous studies have reported the superior insecticidal performance of nano-formulations compared to their conventional counterparts (Table 1). Across diverse AIs and targets, nano-formulations consistently outperform conventional counterparts in lethal efficacy and/or required doses.

For instance, Kumar et al. [116] developed two highly effective fipronil nano-formulations against brown planthoppers (*Nilaparvata lugens*), achieving population suppression rates as high as 93.47%, notably surpassing the 80.47% efficacy of commercial fipronil formulations. Similarly, Zheng et al. [117] evaluated the performance of two emamectin benzoate formulations (0.7% nano-relievers and 5% microemulsions) against *Thrips tabaci*, both of which maintained control efficacy above 90% within 3 to 14 days post-application. Nanometerization of pyrethroids has also shown promise, with synergistic toxicity ratios reaching up to 2-fold against *Culex pipiens* larvae [118]. In another study, the incorporation of cationic nano-chitin whiskers into insecticide significantly increased the mortality of wheat aphids (*Schizaphis graminum*), with corrected mortality exceeding 95% [119]. A broader meta-analysis of publications further confirms the efficacy enhancement of nano-insecticides. Across 314 trials, nano-enabled formulations improved overall control efficacy by an average of 31.5%, while 47 field trials reported an average improvement of 18.9% in real-world conditions [4].

Despite these promising results, current levels of bioactivity enhancement achieved through nanotechnology remain insufficient to fully meet the demands of large-scale agricultural production. Several underlying factors may account for this limitation. Firstly, many nano-insecticide formulations exhibit improved efficacy under laboratory conditions but fail to maintain consistent performance under complicated field environments due to variable temperature, humidity, UV exposure, and crop surface characteristics [120]. Secondly, the lack of unified definitions and regulatory frameworks for nano-insecticides across countries presents a major barrier to commercialization. Without clear criteria, registering nano-enabled insecticides remains difficult and time-consuming [121]. Thirdly, growing concerns about potential biosafety risks and long-term ecological impacts of nano-insecticides may limit their broad adoption, especially in environmentally sensitive agricultural regions [122]. These challenges highlight the urgent need for more robust field validation, standardized regulatory systems, and comprehensive safety assessments to ensure that nano-insecticides can be used as effective components of real-world pest management.

## 6. Environmental Safety of Nano-Insecticides

The rapid development of nano-pesticides has raised growing public and scientific concerns regarding their safety. While nano-formulations offer promising avenues to reduce off-target toxicity and improve insecticide efficacy, they may also introduce novel ecological risks. This section summarizes the current research on the environmental safety of nano-pesticides, focusing on their effects on predatory organisms, beneficial soil microorganisms, and environmental residue behavior. Moreover, it addresses the dual nature of nano-pesticides, highlighting both their advantages and potential drawbacks while proposing possible solutions (Table 2).

### 6.1. Impact on Beneficial Predators

One of the key concerns surrounding nano-insecticides is their possible adverse impact on non-target predatory organisms, such as beneficial arthropods. While enhanced toxicity against pests is desirable, it is crucial to ensure that nano-formulations do not compromise predator populations that contribute to natural pest control. Recent studies have investigated this balance. Yan et al. [143] applied the SPc to deliver cyantraniliprole (CNAP), achieving increased toxicity toward the target pest *Frankliniella occidentalis*. Importantly, although toxicity toward its predator *Orius sauteri* also increases, the selectivity toxicity ratio (STR) rises from 2.33 to 3.23, indicating improved target selectivity. Similar results have been obtained for nanoscale spirotetramat/SPc complexes [144]. However, other studies caution that the oral ingestion of nanocarriers during larval development stages may negatively affect adult longevity, fertility, mobility, and stress tolerance [145]. These findings emphasize the need for chronic toxicity assessments and suggest that optimized nanocarrier design—such as biodegradable or predator-inert formulations—may help mitigate the long-term sublethal effects.

### 6.2. Impact on Beneficial Microorganisms

Beneficial microorganisms play indispensable roles in sustaining soil health and agroecosystem function [146]. Several studies suggest that nano-formulations can reduce toxicity toward soil microorganisms when properly engineered. For example, Balaji et al. [147] developed a hydrodispersive colloidal deltamethrin formulation with reduced toxicity, which exhibited lower inhibition of bacterial growth. Likewise, Janus emulsion systems were reported to alleviate the negative effects of pyraclostrobin on microbial diversity [148]. However, not all nanomaterials are benign. Field trials involving Cu(OH)_2_ nano-pesticides revealed significant alterations in microbial communities: bacterial richness decreases, fungal diversity drops, and community composition shifts over time [149]. These findings underscore the need for safer nanomaterial choices, optimized dosing strategies, and long-term monitoring of soil microbiota.

### 6.3. Environmental Residues

Another critical aspect of nano-insecticide safety is the potential for environmental accumulation and residue persistence. Several publications indicate that nanocarrier systems can reduce insecticide residues in the environment. Darwesh et al. [113] developed insecticide nano-emulsions using nano-chitosan as a natural biopolymer and prepared nano-emulsions that prolonged the residual activity against *S. littoralis* on cotton plants compared to conventional insecticide formulations. Similarly, Jiang et al. [72] and Yan et al. [105] showed that the SPc-based encapsulation of osthole and dinotefuran accelerates the degradation and reduces detectable residues in plant tissues over time. For instance, Cu(OH)_2_ nanoparticles may adsorb thiacloprid, reducing its bioavailability and potentially contributing to residue accumulation [150]. Repeated application of inorganic nanoparticles can also alter soil pH, porosity, and nutrient dynamics, with possible effects on crop performance and soil fauna [151]. While nano-insecticides offer clear environmental advantages—such as reducing application rates and off-target exposure—they also introduce safety concerns that differ from traditional formulations. Balancing efficacy with safety will require the implementation of targeted risk assessments, development of biodegradable or predator-safe nanocarriers, and integration of ecotoxicological testing into regulatory frameworks. Ongoing multidisciplinary collaboration is essential to ensure that nano-pesticides support sustainable agriculture without compromising ecological integrity [147,152].

## 7. Future Perspectives

**Figure 2:**
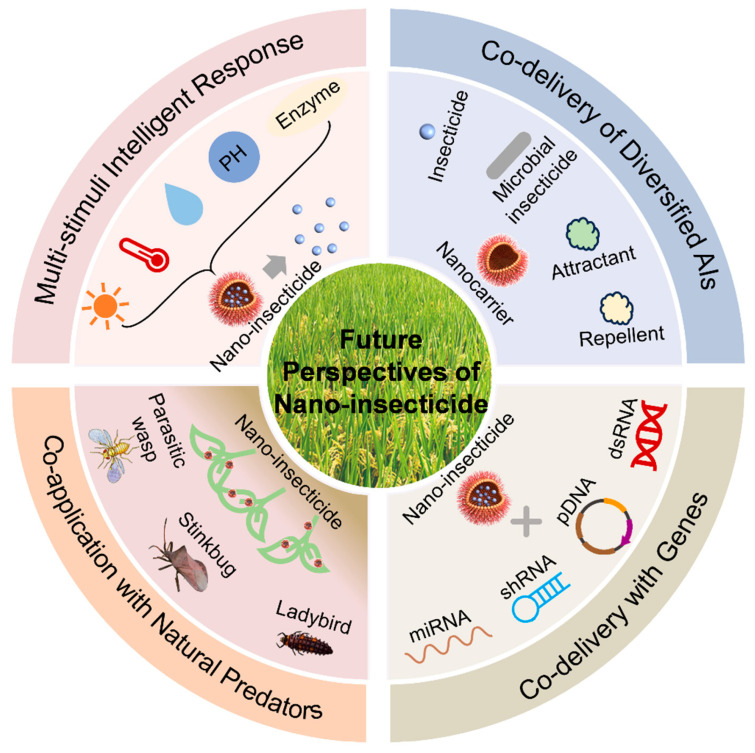
Future perspectives of nano-insecticides for pest management. These integrated strategies can improve the control efficacy and reduce the adverse environmental impact of insecticides.

Nano-insecticides undoubtedly represent a promising frontier in sustainable pest management. However, no single technological platform can permanently meet the dynamic and evolving demands of modern agriculture [6,7,9,12]. To achieve high-quality and eco-friendly agricultural development, interdisciplinary collaboration and the integration of cutting-edge technologies will be critical (Figure 2).

### 7.1. Multi-Stimuli Intelligent Response

To meet the complexity of field conditions, including insect-specific microenvironments, biochemical markers, and pest-secreted enzymes, next-generation nano-pesticides must incorporate intelligent and multi-stimuli-responsive release systems [153,154,155]. Alkaline midguts and lignin-degrading enzymes in phytophagous insects have already been utilized to construct targeted response systems [156]. Lin et al. [155] developed a dual-responsive nano-system sensitive to pH and laccase, enabling the precise and on-demand insecticide release. Similarly, pest-derived molecules, such as glutathione (GSH) and esterase, can serve as biochemical triggers. Shan et al. [157] constructed polymeric nanoparticles with triple-stimuli [153,154,155] responsiveness (GSH, esterase, and alkaline conditions), thereby significantly improving pesticide utilization efficiency. However, the complicated synthesis and high cost of these intelligent systems may limit their large-scale application. Furthermore, ensuring the stability and reliability of these sensitive nanocarriers under variable and harsh field conditions remains a significant technical hurdle.

### 7.2. Co-Delivery of Diversified AIs

Under typical field production conditions, crops are commonly subjected to infestations by diverse insect pests, making a single agent insufficient. Co-delivery systems that incorporate multiple AIs offer synergistic pest control. For example, Zaki et al. [158] combined *Bacillus thuringiensis* with sodium titanate nanoparticles to control cotton leafworm. Limonene and carvacrol were co-encapsulated in zein nanoparticles, achieving enhanced larvicidal effects on *Spodoptera frugiperda* [159]. Furthermore, integrating nanotechnology with semiochemicals (attractants and repellents) shows promise in multifunctional pest control strategies [160,161]. Despite these advantages, developing stable co-delivery formulations can be challenging due to potential physicochemical incompatibilities between different AIs. Moreover, the regulatory approval process for multicomponent nano-formulations presents a considerable barrier to their commercialization.

### 7.3. Co-Delivery with Genes

Combining conventional insecticides with genetic materials, such as miRNA, shRNA, plasmid DNA (pDNA), and double-stranded RNA (dsRNA), has opened new avenues for synergistic pest control. Such systems enhance the efficacy of traditional pesticides while lowering required dosages, revitalizing older compounds and reducing development costs [162]. For instance, gene *nrf2* orchestrates the insect’s xenobiotic defense machinery, enabling neutralization and metabolic detoxification of insecticidal compounds. The dsRNA targeting the detoxification gene *nrf2* can be co-delivered with insecticides to prepare a series of multicomponent nano-insecticides, which can suppress *nrf2* expression to improve the susceptibility of *S. frugiperda* to chlorantraniliprole, emamectin benzoate, and spinetoram [163]. Similarly, Wei et al. [164] developed dsRNA/SPc/botanical insecticide complexes to effectively control aphids. Nevertheless, the practical application of this strategy is currently constrained by the high cost of dsRNA and its inherent instability in the environment. Additionally, potential off-target effects on beneficial organisms and public perception of gene-based technologies require thorough investigation.

### 7.4. Co-Application with Natural Predators

Some nanocarriers (e.g., SPc) have been shown to exhibit no toxicity to the eggs or nymphs of beneficial predators at working concentrations. This low toxicity, along with the enhanced selectivity offered by nano-formulations, can reduce the potential adverse impacts on non-targets, providing a solid basis for their integration with biological control agents [144,165]. For instance, co-application of nano-insecticides (e.g., broflanilide/SPc and chlorobenzuron/SPc) with predatory stinkbugs was reported to increase *Spodoptera litura* mortality by approximately 30% [165,166]. Such integrative strategies not only reduce insecticide input but also support green agriculture and organic production by mitigating environmental impacts. However, it is crucial to conduct further studies on potential sublethal effects, such as impacts on the predator’s reproduction and behavior. The long-term ecological consequences of the nanomaterials themselves, including their potential for bioaccumulation in the food chain, also warrant careful assessment.

## 8. Conclusions

Nano-insecticides offer innovative solutions to several long-standing challenges in crop protection, including low utilization efficiency, pest resistance, environmental pollution, and off-target toxicity. This review comprehensively summarized recent advances in the definition, classification, delivery systems, synergistic mechanisms, efficacy, and safety evaluation of nano-insecticides. The unique physicochemical properties of nano-delivery systems enhance insecticide adhesion, penetration, absorption, and controlled release, ultimately improving pest control outcomes. Furthermore, emerging strategies, such as multi-stimuli responsiveness, co-delivery with genetic materials, and compatibility with biological control agents, have expanded the application potential of nano-insecticides. Despite these advancements, critical challenges remain, including potential biosafety risks, environmental persistence, and the lack of standardized regulatory frameworks. To ensure the responsible deployment of nano-insecticides, future research should prioritize the development of biodegradable, selective, and multifunctional nanomaterials alongside comprehensive long-term safety assessments. Greater interdisciplinary collaboration among materials science, plant protection, and environmental toxicology will be essential. Importantly, the advancement of nano-insecticides should align with global policy priorities, such as the United Nations Sustainable Development Goals (SDGs), the European Union’s Farm to Fork Strategy, and China’s Green Pest Control Action Plan. With continued innovation and responsible governance, nano-insecticides are poised to play a central role in promoting sustainable, high-efficiency, and eco-friendly pest management worldwide.

## Figures and Tables

**Table 1:** Comparative insecticidal efficacy of nano-insecticides vs. conventional insecticides.

Active Ingredient (AI)	Nano-Formulation Type	Target Pest	Nano-Insecticide vs. Traditional Insecticide LC_50_/Mortality	Reference
Type I: nanoscale active ingredients				
Silver (Ag)	Metallic NPs (Ag NPs)	*Spilosoma obliqua*	Nano: LC_50_ 93.21 mg/L; Traditional crude leaf extract: LC_50_ 1590.74 mg/L; Efficiency: 17.07×	[110]
Silica (Si)	Non-metallic NPs (nano-silica)	*Callosobruchus maculatus*	Nano LC_50_: 88.170 mg/L	[111]
CaB_2_O_4_	Non-metallic NPs (sea urchin-like calcium borate microspheres CB-A)	*Spodoptera littoralis*	Nano CB-A: LC_50_ 207 mg/L; Traditional microblocks: LC_50_ 406 mg/L; Efficiency: 1.96×	[112]
Purslane oil	Non-metallic (nano-emulsion)	*Aphis gossypii*	Nano: LC_50_ 72.74 mg/L; Traditional Emulsion: LC_50_ 85.02 mg/L; Efficiency: 1.17×	[26]
Radish oil			Nano: LC_50_ 453.91 mg/L; Traditional Emulsion: LC_50_ 555.42 mg/L; Efficiency: 1.22×	
Rosemary oil			Nano: LC_50_ 72.45 mg/L; Traditional Emulsion: LC_50_ 869.64 mg/L; Efficiency: 12×	
Type II: nanocarrier-loaded insecticides				
Methomyl	Organic carrier (nano-chitosan)	*Spodoptera littoralis*	Nano: LC_50_ 4.97 mg/L; Traditional insecticides: LC_50_ 20.82 mg/L; Efficiency: 4.19×	[113]
β-cyfluthrin	Organic carrier (graphene oxide)	*Ostrinia furnacalis*	Nano: LC_50_ 0.62 mg/L; Traditional insecticide: LC_50_ 1.32 mg/L; Efficiency: 2.13×	[61]
Imidacloprid			Nano: LC_50_ 2.31 mg/L; Traditional insecticide: LC_50_ 4.23 mg/L; Efficiency: 1.83×	
Permethrin	Organic carrier (SLN)	*Artemia salina*	Nano: LC_50_ 3.127 mg/L; Traditional Emulsion: LC_50_ 4.536 mg/L; Efficiency: 1.45×	[114]
*Salvia abrotanoides* extract	Hybrid carrier (Fe_3_O_4_@Carbon)	*Phthorimaea operculella*	Nano: LC_50_ 355.30 mg/L; Traditional pure extract: LC_50_ 660.02 mg/L; Efficiency: 1.86×	[115]

**Table 2:** Comparative environmental impacts: conventional vs. nano-enabled pesticides.

Aspect	Conventional Pesticides	Nano-Enabled Pesticides
Soil residue and degradation	Exhibit low affinity (e.g., Atrazine K_d_ = 0.8 L/kg in sand) [123]. Undergo rapid degradation (e.g., Atrazine DT_50_ = 36 d in sand) [123].	Show enhanced adsorption (e.g., Atrazine, K_d_ = 1.7 L/kg in sand) [123]. Enable nanocarrier controlled release (e.g., Atrazine DT_50_ = 42 d in sand) [123]. Negatively affect non-target soil microorganisms crucial for N, P, and C cycling (e.g., nanoscale Cu(OH)_2_ and Ag) [124]. Reduce adsorption by soil, leading to easy transport via soil pores (e.g., lambda-cyhalothrin-loaded nanogel formulations) [125].
Aquatic pollution risk	Exhibit high leaching potential, particularly neonicotinoids (e.g., chlorpyrifos and tebuconazole) [126]. Demonstrate toxicity to zebrafish (LC_50_ = 0.581 mg/L for Metamifop) [127].	Increase the soil half-life of insecticide by up to 2 times, leading to reduced leaching [126]. Show reduced toxicity to zebrafish (LC_50_ = 1.075 mg/L for Metamifop@HLDP) [127]. Be transported during flush rainfall events (e.g., imidacloprid) [125]. Show higher degradation rates of dimethoate and methomyl using the nanosized ZnO catalyst [128].
Impact on non-target organisms (NTOs)	Harmful effects on bee mouthpart function have been observed with imidacloprid (Mouthparts out and Straight = 45.87%) [129]. Demonstrate lethality to natural enemies (e.g., *Coccinella septempunctaa* exposed to cypermethrin resulted in 90% mortality after 72 h) [130]. Suppress soil microbial activity, as evidenced by a 35.9% reduction in the relative abundance of Acidobacteria due to neonicotinoid insecticides [131].	Significantly reduce bee damage, with nano-imidacloprid showing a lower rate of impaired mouthparts (Mouthparts out and Straight = 16.92%) [129]. Reduce *Coccinella septempunctata* mortality by 15.67% after 72 h [130]. Enrich beneficial microorganisms, leading to a 45.7% increase in the relative abundance of Acidobacteria [131]. Induce oxidative stress in silkworm gut microbiota community clusters [132].
Volatility and atmospheric pollution	Exhibit high volatility (e.g., organophosphates like dichlorvos) [133]. Lead to off-target contamination via spray drift [134].	Volatility can be reduced by silver nanoparticles [133]. Reduce insecticide drift (e.g., CHL@CS/CMCS NP suspension) [134]. Nano-emulsion sprays can generate inhalable aerosols (75–106 nm), thereby increasing the inhalation exposure risk to applicators [135].
Application efficiency and loss	Demonstrate low foliar retention (e.g., 10.03 mg/kg of CHL) [134]. Are susceptible to loss due to rain wash-off (e.g., cyantraniliprole (CTP)) [136]. Exhibit low utilization efficiency [137].	Show high foliar retention (e.g., 12.28 mg/kg of CHL@CS/CMCS NP) [134]. Provide resistance to rain erosion (e.g., CTP-PLA MS and CTP-CaCO_3_ MS) [136]. Improve the efficiency of transportation and conduction of AIs [137]. High production costs can limit scalability (e.g., MOF carriers) [138].
Bioaccumulation and long-term risk	Exhibit high bioconcentration factors (e.g., conventional copper salts accumulating up to 105 μg Cu per g dry mass) [139]. Metabolites may show genotoxicity (e.g., dinotefuran) [140].	Demonstrate lower bioconcentration factors (e.g., conventional copper salts accumulating 55 μg Cu per g dry mass) [139]. Reduce insecticide residue (e.g., dinotefuran/SPc formulation) [140]. Accumulate in the crucial intestinal flora of honeybees (e.g., O-CMC-NPs) [141]. GO can enhance the bioconcentration of cis-BF [142].
